# Unchanged PCNA and DNMT1 dynamics during replication in DNA ligase I-deficient cells but abnormal chromatin levels of non-replicative histone H1

**DOI:** 10.1038/s41598-023-31367-4

**Published:** 2023-03-16

**Authors:** Seema Khattri Bhandari, Nathaniel Wiest, Annahita Sallmyr, Ruofei Du, Laure Ferry, Pierre-Antoine Defossez, Alan E. Tomkinson

**Affiliations:** 1 Cancer Research Facility, Departments of Internal Medicine and Molecular Genetics & Microbiology, University of New Mexico Comprehensive Cancer Center, University of New Mexico Health Sciences Center, 915 Camino de Salud, 1 University of New Mexico, Albuquerque, NM 87131 USA; 2 Epigenetics and Cell Fate, CNRS, Université Paris Cité, 750013 Paris, France; 3 Present Address: Division of Hematology and Medical Oncology, Department of Internal Medicine, Mayo Clinic, Jacksonville, FL 32224 USA; 4 Present Address: Department of Biostatistics, University of Arkansas for Medical Sciences, Little Rock, AR 72205 USA

**Keywords:** Biochemistry, Biological techniques

## Abstract

DNA ligase I (LigI), the predominant enzyme that joins Okazaki fragments, interacts with PCNA and Pol δ. LigI also interacts with UHRF1, linking Okazaki fragment joining with DNA maintenance methylation. Okazaki fragments can also be joined by a relatively poorly characterized DNA ligase IIIα (LigIIIα)-dependent backup pathway. Here we examined the effect of LigI-deficiency on proteins at the replication fork. Notably, LigI-deficiency did not alter the kinetics of association of the PCNA clamp, the leading strand polymerase Pol ε, DNA maintenance methylation proteins and core histones with newly synthesized DNA. While the absence of major changes in replication and methylation proteins is consistent with the similar proliferation rate and DNA methylation levels of the *LIG1* null cells compared with the parental cells, the increased levels of LigIIIα/XRCC1 and Pol δ at the replication fork and in bulk chromatin indicate that there are subtle replication defects in the absence of LigI. Interestingly, the non-replicative histone H1 variant, H1.0, is enriched in the chromatin of LigI-deficient mouse CH12F3 and human 46BR.1G1 cells. This alteration was not corrected by expression of wild type LigI, suggesting that it is a relatively stable epigenetic change that may contribute to the immunodeficiencies linked with inherited LigI-deficiency syndrome.

## Introduction

Half the eukaryotic nuclear genome is duplicated in a discontinuous cyclical manner at the replication fork. During lagging strand synthesis, millions of short RNA–DNA primers are initially synthesized by the DNA Polymerase α (Pol α)-primase complex^1^. The Proliferating Cell Nuclear Antigen (PCNA) sliding clamp protein is then loaded at the primer terminus by Replication Factor C (RFC) to serve as a platform for coordination of gap filling DNA synthesis by DNA Polymerase δ (Pol δ), processing of the 5’end of the adjacent Okazaki fragment by Flap Endonuclease 1 (FEN-1), and the joining of Okazaki fragments by DNA ligase I (LigI)^1^. Finally, the PCNA clamp is unloaded for subsequent cycles of Okazaki fragment synthesis, processing, and joining^1–3^. While the recruitment of LigI to replication foci is largely dependent upon its interaction with PCNA^4,5^, LigI also interacts with Pol δ within a functional PCNA-Polδ-FEN-1 complex and then remains associated with the DNA after ligation in a complex with PCNA^6^. In *S*. *cerevisiae*, PCNA unloading by Elg1-RFC is dependent upon the joining of Okazaki fragments by the LigI homolog Cdc9^7^, suggesting that human Elg1-RFC homolog, ATAD5-RFC, may recognize and unload the LigI-PCNA complex^7–9^. However, mammalian cells are able to utilize the DNA ligase IIIα (LigIIIα)/XRCC1 complex, which is not present in *S. cerevisiae*, to join Okazaki fragments in the absence of LigI^10,11^. While an interaction between XRCC1 and PCNA has been described^12^, there is limited information about how the lagging strand is replicated to generate an intact strand in Lig1-deficient cells.

There is mounting evidence indicating that lagging-strand DNA synthesis at the replication fork is also intricately linked with maintenance DNA methylation and chromatin restoration^13–15^. For example, LigI not only interacts with the replication factors PCNA, RFC and Pol δ, but also with Ubiquitin Like With PHD And Ring Finger Domains (UHRF1) in a methylation-dependent manner^15,16^. Interestingly, this interaction involves a histone H3K9-like mimic within LigI and serves to recruit UHRF1 and DNA methyltransferase 1 (DNMT1) to replication sites to promote maintenance DNA methylation by DNMT1^15,16^. It is, however, not known how lagging strand DNA replication is coordinated with DNA methylation and chromatin assembly in cells deficient in LigI. As human *LIG1* deficiency syndromes have been described^17–19^, and LigI is a promising target in ovarian cancer^20^, the mechanisms of lagging-strand DNA synthesis and associated DNA methylation and histone deposition dynamics in LigI deficient cells are relevant to understanding disease pathogenesis and the potential utility of LigI inhibition for treating cancer.

Here, we examined protein association with and dissociation from newly synthesized DNA in wild-type and LigI deficient cells. Surprisingly, we found that LigI deficiency did not alter PCNA turnover in human or mouse LigI-deficient cells. Additionally, both nucleosome assembly and DNA methylation machinery recruitment were intact in *LIG1* null cells. However, linker histone composition was altered as there was an enrichment of a non-replicative version of histone H1, histone H1.0, on both newly synthesized DNA and bulk chromatin^21^. This occurred in both mouse *LIG1* null^22^ and human *lig1* mutant cells with reduced levels of DNA ligase I activity^17^ and was not corrected by expression of wild type LigI, suggesting that the alteration in chromatin histone H1 composition reflects a stable epigenetic change in response to LigI-deficiency. Together our results provide novel insights into the role of Lig1 in the dynamics of lagging-strand replication proteins, DNA methylation machinery, and histone deposition.

## Results

### Effect of DNA ligase I-deficiency on the association of proteins involved in Okazaki fragment synthesis and processing with newly synthesized DNA

**Figure 1:**
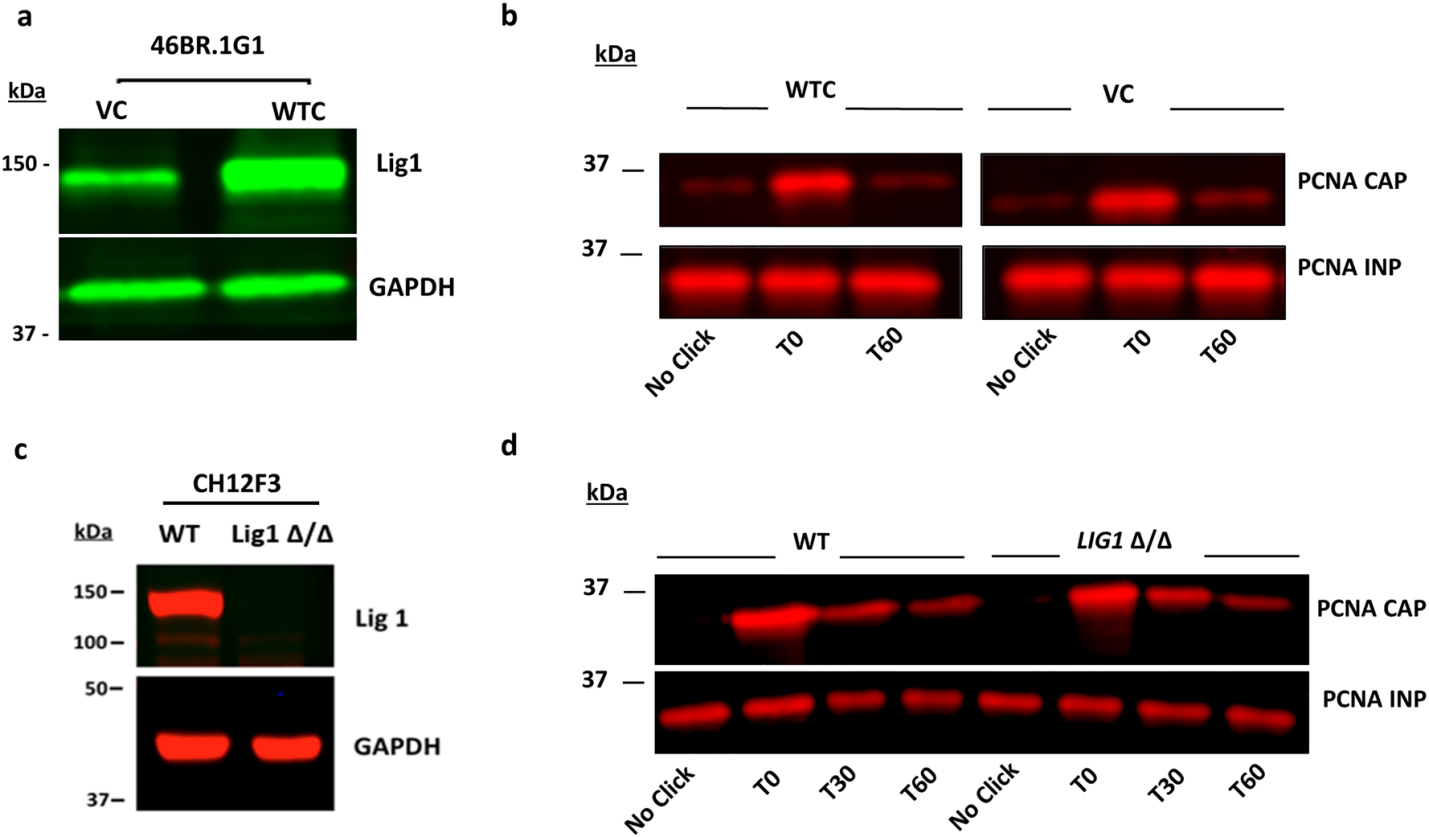
LigI is not essential for PCNA deposition on and unloading from replicating DNA. DNA ligase I and GAPDH detected by immunoblotting in extracts (50 μg) from; **(a)** human LigI deficient fibroblast cell line 46BR.1G1 (VC) and a derivative stably expressing wild type LigI (WTC) and; **(c)** mouse B cell line CH12F3 (WT) and a *LIG1* null derivative (Δ/Δ). The human **(b)** and mouse **(d)** cell lines were processed by aniPOND and immunoblotted for PCNA. CAP = 50% of beads capture proteins. INP = 1% of pre-pulldown proteins. Samples indicated as T0, T30, and T60 correspond to a 15 min EdU pulse followed by 0, 30, and 60 min thymidine chase, respectively. Uncropped Western blots are shown in supplementary Fig S6.

To identify alterations in the kinetics of proteins associated with newly synthesized DNA in LigI-deficient cells, we employed isolation of proteins on nascent DNA (iPOND)^23^ and a modified version of iPOND, accelerated native iPOND (aniPOND), in which proteins associated with EdU-containing DNA in native rather than cross-linked chromatin are affinity purified^24^. In initial studies using aniPOND, we compared the kinetics of PCNA association with and dissociation from newly synthesized DNA in *lig1* mutant human fibroblasts, 46BR.1G1, that have reduced steady state levels of a catalytically defective LigI with a derivative of 46BR.1G1 cells stably expressing wild type LigI (Fig. 1a)^17,25,26^. As shown in Fig. 1b, LigI deficiency did not result in alterations in either PCNA association with or turnover from newly synthesized DNA as measured by quantitative near-infrared immunoblotting. Similar results were obtained with wild type and *LIG1* null versions of the mouse B-cell lymphoma cell line, CH12F3 (Fig. 1c,d)^22^. Thus, we conclude that LigI is not required for the efficient turnover of PCNA at the replication fork in mammalian cells.

**Figure 2:**
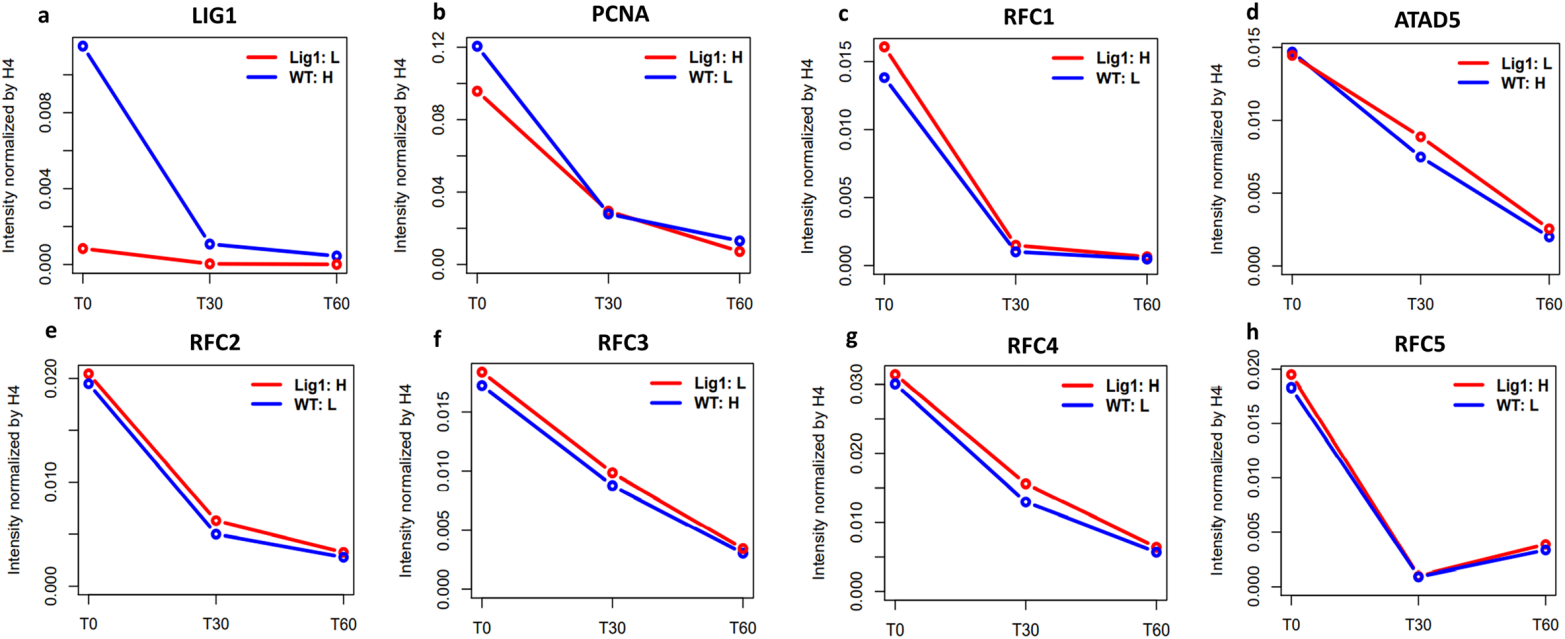
The absence of LigI does not alter the kinetics of PCNA, the PCNA loader (RFC1) and the PCNA unloader (ATAD5) association with newly synthesized DNA. CH12F3 wild type cells and a *LIG1* null derivative grown in media containing light (WT:L, blue line) or heavy (LigI:H, red line) isotope labeled amino acids, respectively, were pulse labeled with EdU for 15 min followed by incubation with thymidine containing media for 0 (T0), 30 (T30) and 60 (T60) min. After processing by aniPOND, the relative levels of; **(a)** LigI; **(b)** PCNA; **(c)** RFC1; **(d)** ATAD5; **(e)** RFC2; **(f)** RFC3;** (g)** RFC4; and **(h)** RFC5 associated with EdU-labeled DNA were determined by mass spectrometry as described in Materials and Methods.

**Figure 3:**
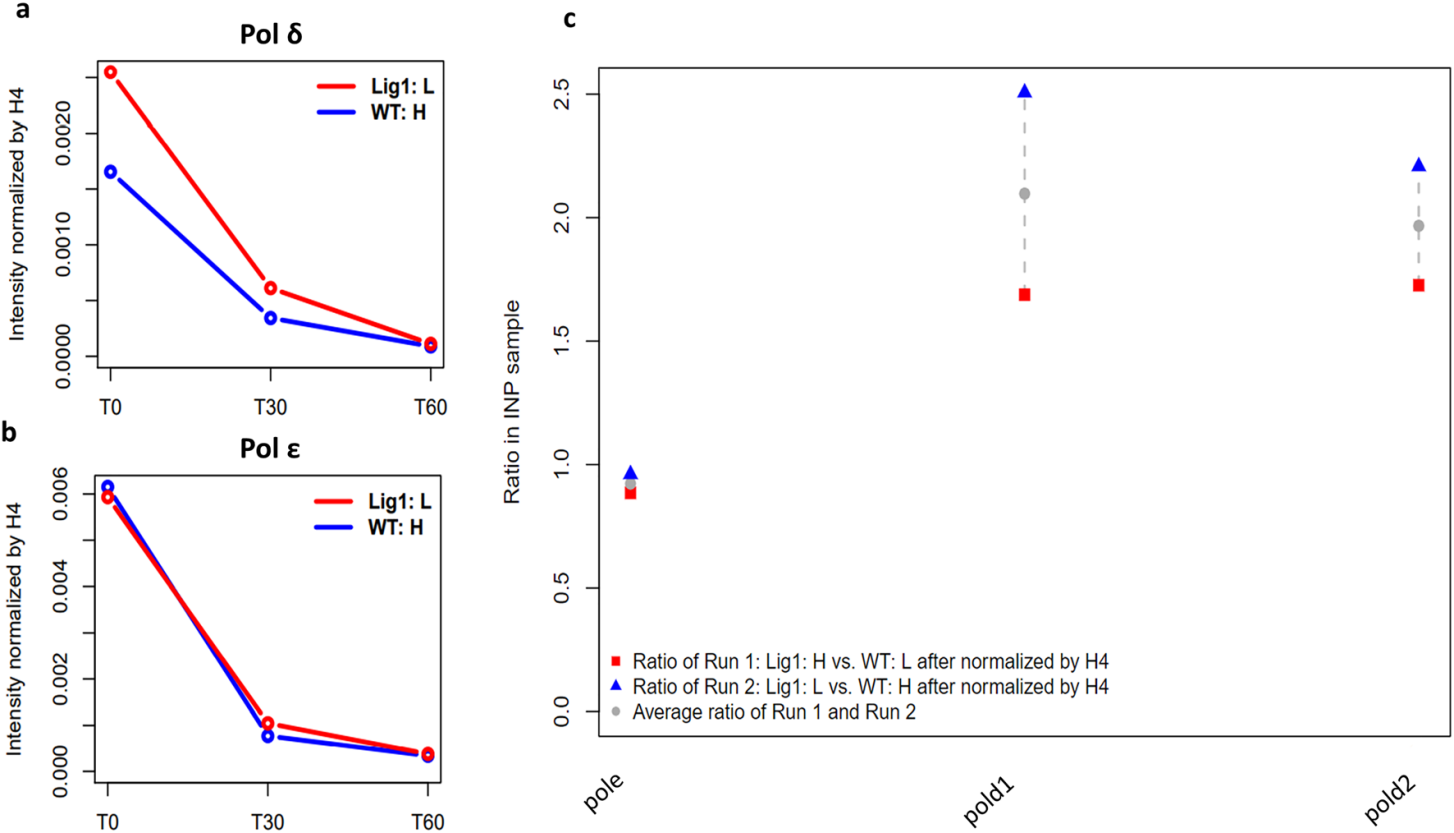
Increased association of DNA Pol δ with replicating DNA and chromatin in *LIG1* null cells. The relative levels of; **(a)** Pol δ and **(b)** Pol ε associated with EdU-labeled DNA in CH12F3 wild type cells (WT:L, blue line) and a *LIG1* null derivative (LigI:H, red line) were determined by mass spectrometry as described in Materials and Methods. (**c)** The levels of Pol ε, Pol δ1, and Pol δ2 in the bulk chromatin are shown as a ratio of *LIG1* null to WT cells in two independent experiments (red square and blue triangle) and their average (grey dot).

To enhance the detection and quantitation of proteins associated with EdU-DNA in native chromatin, wild type and *LIG1* null mouse B-cells were grown in media containing amino acids labeled with different stable isotopes followed by mass spectrometry (SILAC-MS) to identify proteins and determine their relative amounts in the cell lines^27^. To confirm that differences in protein amounts were not due to the different labeling media, a second SILAC-MS experiment was performed in which the labeling media was switched (Fig. S1). As expected, this approach confirmed the absence of LigI in *LIG1* null cells (Fig. 2a and Fig. S1a) and that, even in the absence of LigI, PCNA association with or turnover from newly synthesized DNA was not altered (Fig. 2b and Fig. S1b). Similarly, there were no differences in the behavior of RFC1 and ATAD5 (Fig. 2c,d, and Fig. S1c,d), the large unique subunit of the PCNA clamp loader and unloader respectively, as well as the four common RFC small subunits (Fig. 2e–h and Fig. S1e–h)^3^. We did not detect either FEN-1 or the two small subunits of RPA bound to the newly synthesized DNA in the SILAC-MS experiments. *LIG1* null cells had similar steady state levels of PCNA, ATAD5, and RFC1-5 proteins associated with bulk chromatin compared with wild type cells (Fig. S2). Interestingly there was increased association of the lagging strand DNA polymerase, Pol δ, with newly synthesized DNA (Fig. 3 and Fig. S1i) and, to a greater extent, with the bulk chromatin (Fig. 3c) of *LIG1* null cells whereas the absence of LigI did not alter the steady state levels of leading strand DNA polymerase, Pol ε, associated with either newly synthesized DNA or bulk chromatin (Fig. 3b,c and Fig. S1j).

### Association of DNA ligase III $\boldsymbol{\alpha }$/XRCC1 with replicating DNA in ligase I deficient cells

**Figure 4:**
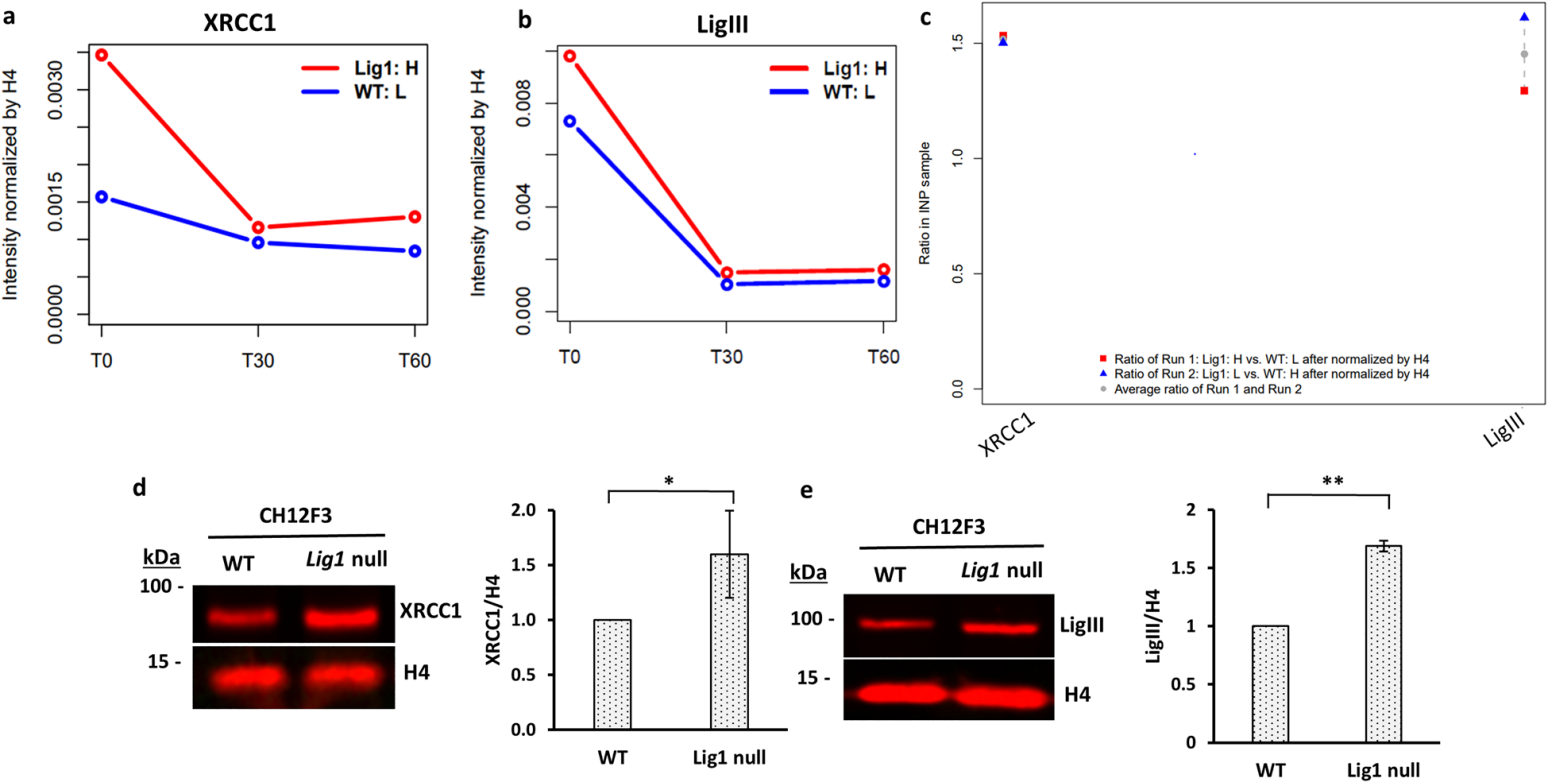
Increased association of XRCC1 with replicating DNA and higher levels of chromatin bound XRCC1 and LigIIIα in *LIG1* null cells. The relative levels of (**a**) XRCC1 and (**b**) LigIIIα associated with EdU-labeled DNA in CH12F3 wild type cells (WT:L, blue line) and a *LIG1* null derivative (LigI:H, red line) were determined by mass spectrometry as described in “Materials and methods”. **(c)** The levels of XRCC1 and LigIIIα in the bulk chromatin are shown as a ratio of *LIG1* null to WT cells in two independent experiments (red square and blue triangle) and their average (grey dot). Immunoblotting of; **(d)** XRCC1 and; **(e)** LigIIIα in chromatin fractions from CH12F3 wild type cells and a *LIG1* null derivative. Histone H4 immunoblot is shown as a loading control for chromatin. Data shown graphically are the mean ( ±) SEM of three independent experiments. Unpaired two-tailed Student test. ‘*’ P-values < 0.05, ‘**’ = P-values < 0.01. Uncropped Western blots are shown in supplementary Fig S7.

To provide further support that DNA ligase IIIα (LigIIIα) functions as the backup DNA ligase in Lig1-deficiency states^10,11,28^, we assessed the levels of both LigIIIα and its binding partner XRCC1 on replicating DNA and bulk chromatin in *LIG1* null CH12F3 mouse cells. As expected, increased association of XRCC1 with newly synthesized DNA and bulk chromatin of *LIG1* null cells was detected by SILAC-MS (Fig. 4a,c and Fig. S1o). The presence of higher levels of chromatin-bound XRCC1 in *LIG1* null cells was confirmed by immunoblotting of chromatin fractions (Fig. 4d). While elevated levels of LigIIIα associated with newly synthesized DNA in the *LIG1* null cells were detected in only one of the SILAC-MS experiments (Fig. 4b), higher levels were observed in bulk chromatin (Fig. 4c) and by immunoblotting of chromatin fractions from *LIG1* null cells (Fig. 4e). Since the total steady state levels of XRCC1 and LigIIIα protein (Fig. S3) and mRNA (Table S1) were similar in wild type and *LIG1* null mouse B-cells, our findings support the conclusions that, in the absence of LigI, the LigIIIα-XRCC1 complex is recruited to replication forks and joins Okazaki fragments^10,11^.

### Efficient recruitment of the maintenance DNA methylase to newly synthesized DNA in the absence of DNA ligase I

**Figure 5:**
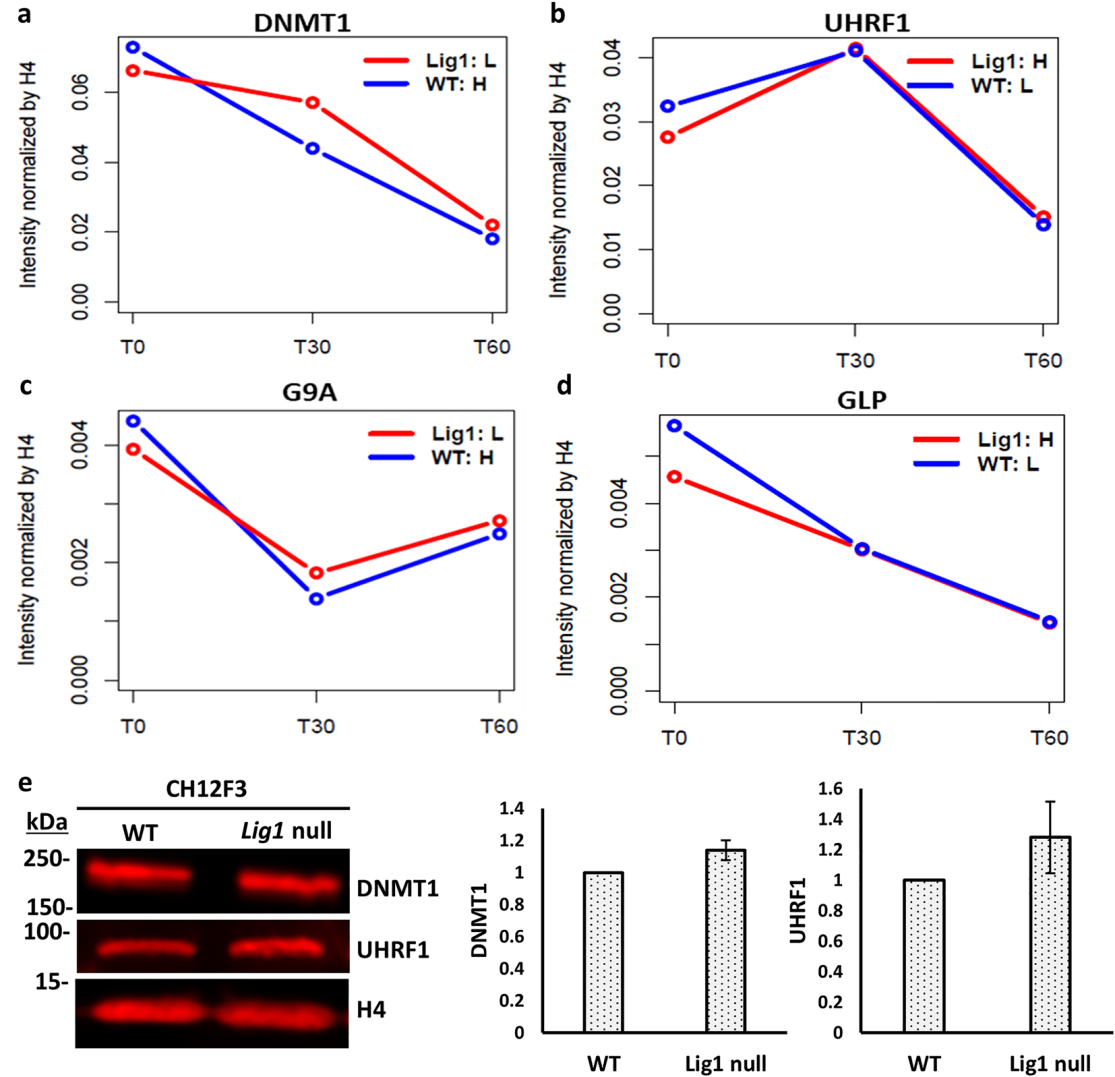
Absence of ligase I does not alter the kinetics of DNMT1, UHRF1, G9A and GLP proteins on replicating DNA. The relative levels of; **(a)** DNMT1; **(b)** UHRF1; **(c)** G9A; and **(d)** GLP associated with EdU-labeled DNA in CH12F3 wild type cells (WT: L, blue line) and a *LIG1* null derivative (LigI:H, red line) were determined by mass spectrometry as described in “Materials and methods”. (**e)** Immunoblotting of DNMT1, UHRF1 and histone H4 in chromatin fractions from CH12F3 wild type cells and a *LIG1* null derivative. Data shown graphically are the mean ( ±) SEM of three independent experiments. Uncropped Western blots are shown in supplementary Fig S8.

Recently it was shown that LigI has a TARK motif identical to the TARK motif in histone H3 that directs methylation of lysine 9^15,16^. In LigI, the lysine residue in the TARK motif is methylated by the enzymes G9a and GLP (Ehmt2 and Ehmt1 in mice) with the interaction between the Tandem Tudor domain (TTD) of UHRF1 and the methylated TARK motif of LigI, recruiting UHRF1 and its interacting partner protein, DNMT1 to sites of replication^15,16^. Surprisingly, while the recruitment of DNMT1 and UHRF1 to the replication sites was not detectable in *LIG1* null cells, these cells had normal levels of DNA methylation^15^. In accordance with this observation, the absence of LigI did not result in alterations in DNMT1, UHRF1, G9a, or GLP recruitment to and dissociation from newly replicated DNA (Fig. 5a–d and Fig. S1k–n) and similar levels of these proteins were associated with bulk chromatin in WT and *LIG1* null CH12F3 cells (Fig. S2). Immunoblotting of chromatin fractions of WT and *LIG1* null cells also confirmed similar levels of DNMT1 and UHRF1 (Fig. 5e). Thus, the methylation of newly replicated DNA by DNMT1 is not dependent upon LigI, providing further evidence that there are functionally redundant recruitment mechanisms for recruitment of the maintenance DNA methylation machinery to newly replicated DNA^29,30^.

### Chromatin assembly and maturation in *LIG1* null cells

**Figure 6:**
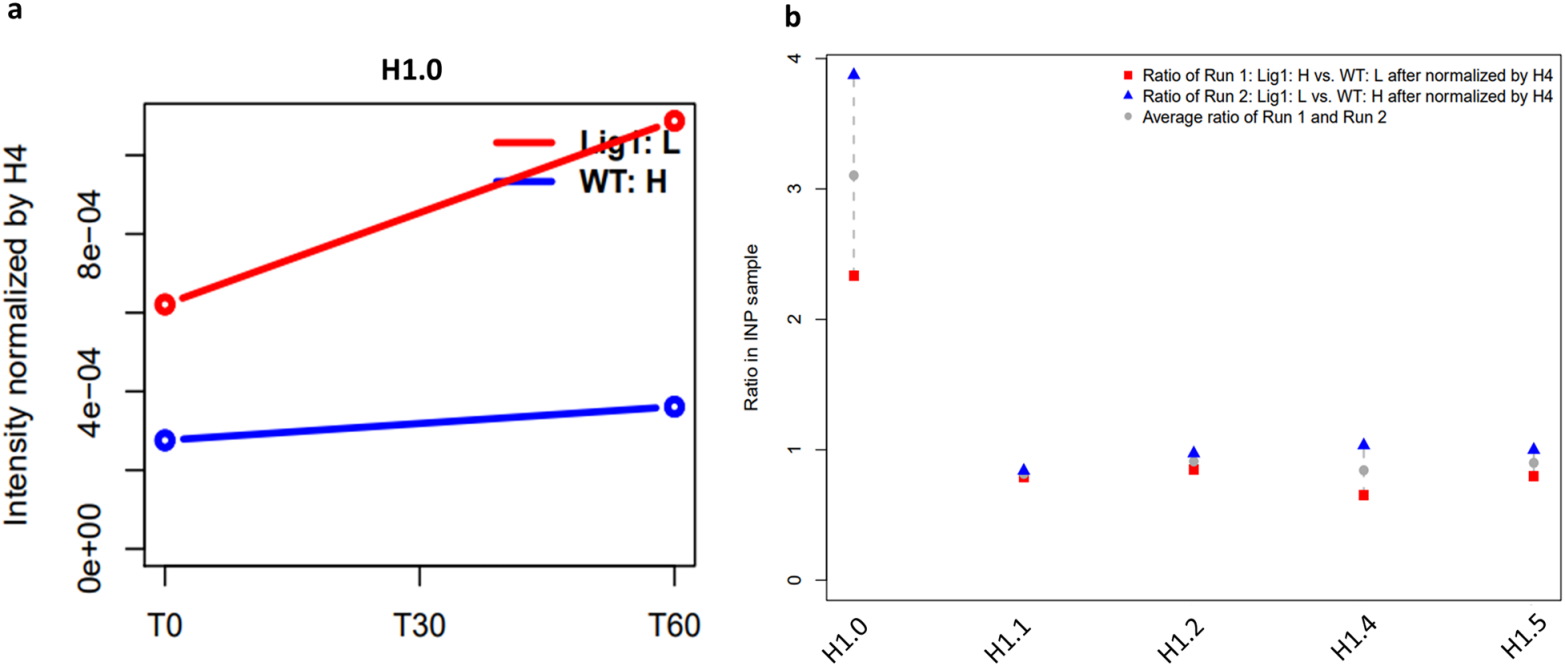
Increased association of H1.0 with replicating DNA and chromatin in *LIG1* null cells. **(a)** The relative levels of H1.0 associated with EdU-labeled DNA in CH12F3 wild type cells (WT: L, blue line) and a *LIG1* null derivative (LigI:H, red line) were determined by mass spectrometry as described in “Materials and methods”. (**b)** The levels of histone H1 variants (H1.0, H1.1, H1.2, H1.4, H1.5) in the bulk chromatin are shown as a ratio of *LIG1* null to WT cells in two independent experiments (red square and blue triangle) and their average (grey dot).

**Figure 7:**
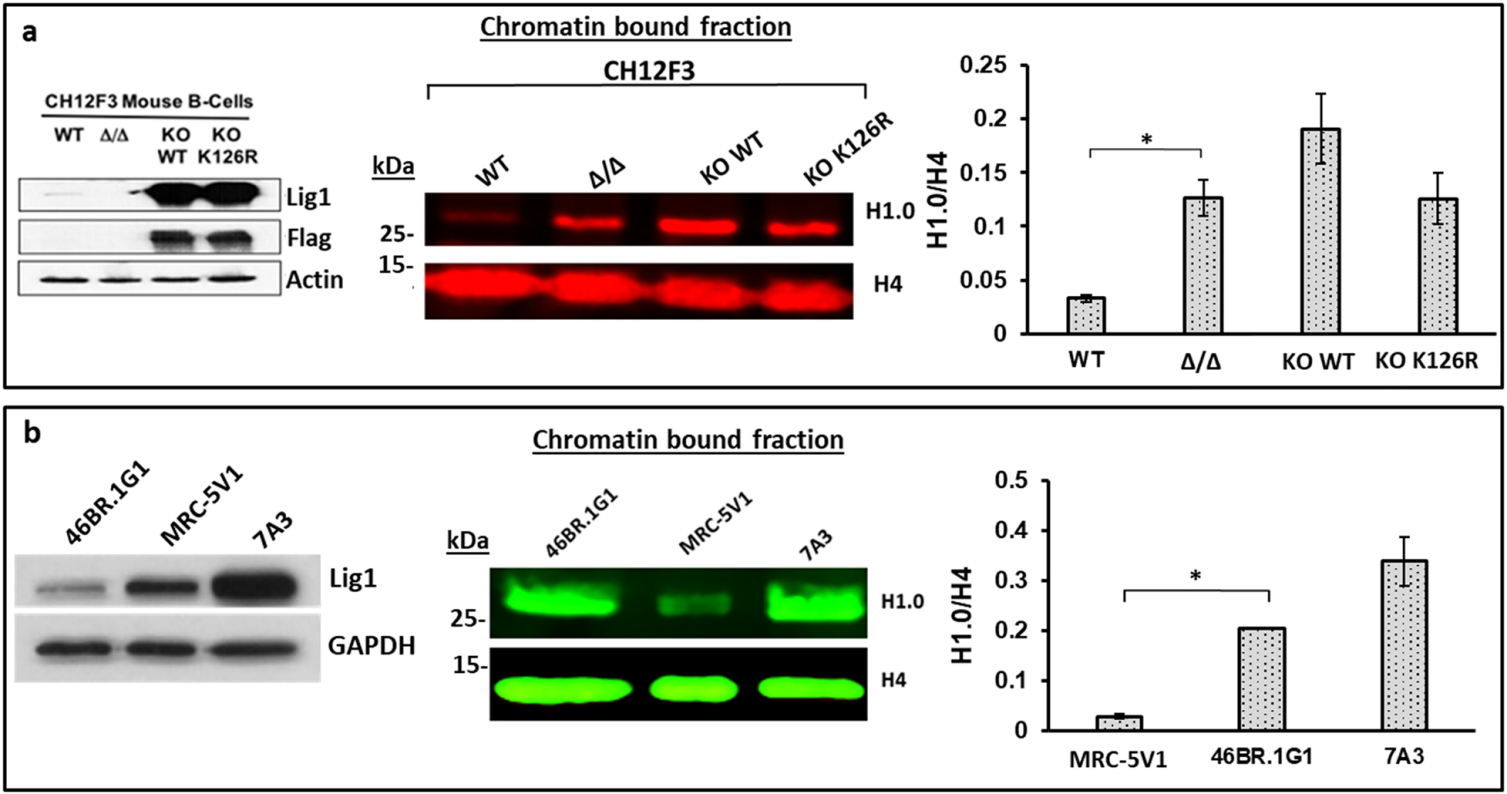
Increased levels of chromatin bound H1.0 in Ligase I deficient cells. (**a)** Immunoblots of whole cell extracts (50 μg, left panel) and chromatin fractions (50 μg, middle panel) from CH12F3 wild type cells (WT) and a *LIG1* null derivative (Δ/Δ) as well as the *LIG1* null cells expressing either Flag-tagged wild type LigI (KO WT) or the TARK mutant version (KO K126R) with the indicated antibodies. (**b)** Immunoblots of whole cell extracts (50 μg, left panel) and chromatin fractions (50 μg, middle panel) from LigI-deficient 46BR.1G1 cells (46BR.1G1), a complemented derivative of 46BR.1G1 cells stably expressing wild type LigI (7A3) and a comparable wild type human fibroblast (MRC-5V1). Data shown graphically are the mean ( ±) SEM of three independent experiments. Unpaired two-tailed Student test. ‘*’ P-values < 0.05. Uncropped Western blots are shown in supplementary Fig S9.

There were no significant variations in the deposition of core histone protein H4 in *LIG1* null mouse B-cells (Fig. S4a). Levels of the histone variants H2AX and H3.3 were also unaltered on newly synthesized DNA (Fig. S4c,d), indicating that the assembly of nucleosomes is not dependent upon LigI. There were, however, higher steady state levels of the replication-independent histone H1 variant, H1.0 on newly synthesized DNA (Fig. 6a and Fig. S4b) as well as bulk chromatin in the absence of LigI^21^. In contrast, the levels of replication dependent variants of linker histone H1 (H1.1, H1.2, H1.4 and H1.5)^21^ were slightly decreased (Fig. 6b), possibly reflecting the increased occupancy of H1.0. The increased steady state levels of H1.0 in the chromatin of the mouse *LIG1* null cells was confirmed by immunoblotting (Fig. 7a). Similarly, isolates of the LigI-deficient human fibroblast cell line 46BR.1G1 from the Montecucco^31^ (Fig. 7b) and the Tomkinson^25,26,32^ laboratories (Fig. S5) also had elevated levels of chromatin H1.0 compared with comparable human fibroblast cell lines, MRC-5V1 and GM00847, respectively that express endogenous wild type LigI^26,31,32^. While the level of *H1F0* mRNA was, unlike the mRNAs encoding proteins involved in DNA replication and maintenance methylation slightly elevated (~ 1.6-fold higher) in *LIG1* null cells (Table S1), the total steady levels of histone H1.0 protein were similar in wild type and *LIG1* null mouse B-cells (Fig. S3). Surprisingly, expression of wild type LigI or the non-methylatable TARK mutant version of LigI in *LIG1* null mouse cells or wild type DNA ligase I in human 46BR.G1 cells did not reduce the levels of chromatin-associated H1.0 to those observed in the parental CH12F3 or comparable normal human fibroblasts, respectively (Fig. 7a,b, Fig. S5). Thus, our results indicate that the chromatin levels of H1.0 are specifically increased in LigI-deficient cells and suggest that this increase reflects a stable epigenetic change.

## Discussion

A better understanding of the consequences of LigI deficiency is becoming increasingly relevant to human health given the recognition of an inherited LigI-deficiency syndrome^17–19^ and the exploration of DNA ligase inhibitors as therapeutic agents^20,33–37^. Here, we have exploited the viability of *LIG1* null mouse and *lig1* mutant human cells to examine the effect of LigI deficiency on replication and DNA methylation protein dynamics on newly synthesized DNA as well as chromatin assembly. We utilized proteomic approaches that allow quantitative analysis of replication protein recruitment and dissociation with newly synthesized DNA^23,24,27^. Our results provide the most in-depth analysis on the effects of LigI deficiency on protein dynamics at the replication fork to date.

It has been shown that Okazaki fragment ligation by *S. cerevisiae* Cdc9 is intrinsically coupled with both PCNA unloading and nucleosome assembly^7,8^. However, these studies did not provide insights as to the contributions of protein–protein interactions involving Cdc9 compared with Cdc9 catalytic activity. In contrast to *S. cerevisiae*, mammals have an additional DNA ligase encoded by the *LIG3* gene that can act as a backup, joining Okazaki fragments in the absence of LigI^10,11,22,28^. Our studies have revealed that the absence of LigI does not alter PCNA association with or turnover from newly synthesized DNA. Since the PCNA sliding clamp plays a key role in coordinating the assembly of nucleosomes on newly synthesized DNA at the replication fork as well as the synthesis, processing, and joining of Okazaki fragments^1,29,30,38–41^, it is likely that that the normal recruitment of core histones reflects the unchanged dynamics of PCNA at the replication fork in LigI-deficient mammalian cells. While the normal levels of DNA methylation in *LIG1* null cells were surprising, given the reduced recruitment of DNMT1 to replication foci^15^, it is possible that this is due to robust replication-uncoupled maintenance methylation^42^. Our results showing that the kinetics of DNMT1 association with newly synthesized DNA were not affected by the absence of LigI and were similar to those of PCNA provide evidence that replication-coupled recruitment of DNMT1 is not dependent upon LigI and suggest that replication foci may represent localized high concentrations of replication proteins from which replication forks are assembled. While there is a PCNA-dependent mechanism to recruit DNMT1 to replicating chromatin in addition to the interaction between UHRF1 and methylated LigI^15,29,30^, the interaction with PCNA is not essential for maintaining methylation levels^43,44^. Since expression of the LigI TARK mutant that retains PCNA binding resulted in reduced methylation levels^15^, it appears that there may be competition between the LigI-UHRF1-DNMT1 and the PCNA-DNMT1 recruitment mechanisms.

Consistent with studies showing higher levels of XRCC1, the partner protein of LigIIIα^45–47^, co-localizing with replication foci in LigI-deficient cells^10,48^, elevated levels of XRCC1 were associated with newly synthesized DNA and both XRCC1 and LigIIIα were present at higher levels on bulk chromatin in the *LIG1* null cells, despite the similar total steady levels of XRCC1 and LigIIIα protein (Fig. S3) in wild type and *LIG1* null cells. Notably, the kinetics of association and release of XRCC1 were similar to those of PCNA and other replication proteins, suggesting that the LigIIIα/XRCC1 complex is specifically recruited to and physically and functionally associates with the replication machinery in the absence of LigI, possibly via an interaction between XRCC1 and PCNA^12^. Since the joining of Okazaki fragment is likely to be the signal for PCNA unloading^7,8^, our results showing that LigI-deficiency does not result in a detectable change in the kinetics of PCNA association with and release from newly synthesized DNA suggests that LigIIIα/XRCC1 is able to effectively join Okazaki fragments. However, since both LigI-deficient^31,48^ and *LIG1* null cells (data not shown) have elevated levels of poly (ADP-ribose) and γH2AX, indicative of higher steady state levels of single- and double-strand breaks, respectively, it appears that LigIIIα/XRCC1 cannot fully substitute for LigI in lagging strand synthesis. The elevated levels of the lagging strand DNA polymerase Pol δ but not the leading strand polymerase Pol ε with both newly synthesized DNA and bulk chromatin is consistent with a specific defect in lagging strand synthesis and previous reports describing a delay in the conversion of Okazaki fragments into high molecular weight DNA^32,49^. Interestingly, LigI-deficiency results in increased incorporation of thymidine into acid soluble DNA^32^ and increased replication fork speed^50^, suggesting that LigI deficiency may result in an increase in Okazaki fragment length and/or displacement of unligated Okazaki fragments by Pol δ. Further studies are needed to more precisely determine the alteration in lagging strand synthesis caused by LigI deficiency.

In S. *cerevisiae*, nucleosome deposition on the adjacent Okazaki fragment appears to determine the extent of DNA synthesis and 5’ end processing that generate a ligatable nick^51^. Our results showing that the deposition of core histones was not affected by the absence of LigI are consistent with a similar mechanism in which assembly of the nucleosome core precedes ligation. There was, however, a surprising change in the composition of the linker histone H1. *LIG1* null mouse cells as well as LigI-deficient human fibroblasts had markedly higher levels of the replication-independent H1 variant, H1.0, in their chromatin whereas there were small decreases in the replication-dependent H1 variants H1.1, H1.2, H1.4 and H1.5^21,52^. Surprisingly, complementation of the *LIG1* null mouse and Lig1-deficient human cells with wild-type Lig1 did not result in a reduction in H1.0 levels, suggesting that Lig1-deficient cells undergo a stable change in linker histone composition. Further studies are needed to determine whether LigI-deficiency effects other cell types in a similar manner. While higher H1.0 levels are typically found in fully differentiated cells^53^, there is evidence that the role of H1.0 levels in cancer may depend on the cell type. The observations that tumor cells with downregulated H1.0 have long term self-renewal capacity and that increasing H1.0 levels reduces the tumorigenic potential of cancer cells^53^ are consistent with an inverse relationship between H1.0 levels and the growth properties of the cancer cell. However, elevated H1.0 levels appear to confer resistance to paclitaxel and correlate with disease recurrence and poor survival in ovarian cancer^54^, suggesting that there may be high levels of H1.0 in a non-dividing or slow growing subpopulation of cancer cells that are resistant to chemotherapy. In contrast to H1.0, the steady state levels of LigI are often elevated in cancer cell lines and tumor samples^20,55^ but low in non-proliferating and differentiated cells^56,57^. Thus, it is possible that reduced levels of LigI serve as a differentiation signal that results in up-regulation of histone H1.0 and may contribute to non-reversible epigenetic changes.

Together our results demonstrate that there are no detectable changes in the dynamics of PCNA and DNMT1 association and dissociation with the replication fork when Okazaki fragments are joined by the LigIIIα-dependent backup-pathway that is required for cell viability in the absence of LigI^10,22,28^. It is possible that the XRCC1 subunit of the LigIIIα/XRCC1 complex interacts with the PCNA clamp remaining at the nick between unjoined Okazaki fragments^6^, coordinating ligation with PCNA unloading. The relatively minor effect of the absence of LigI on protein dynamics at the replication fork is consistent with the cytostatic rather than cytotoxic activity of a LigI inhibitor^34^, suggesting that a LigI inhibitor will have limited toxic side-effects on normal tissues and cells but may have utility in the selective targeting of cancer cells with specific DNA repair defects^20^. Since chemical inhibition and genetic loss can cause different phenotypes^58^, further studies analyzing the acute effects of Lig1 chemical inhibition on replication protein dynamics are needed. While an inherited LigI deficiency syndrome has been described^17–19^, it is difficult to reconcile the defects in DNA replication and repair caused by LigI deficiency with the spectrum of immune deficiencies associated with this syndrome. Our finding that LigI deficiency may cause a epigenetic change in terms of altered composition of the H1 linker histones provides a possible alternative mechanism by which LigI deficiency could impact immune system function.

## Methods

### Cell culture

Wild type and *LIG1* null CH12F3 cell lines were generated as previously described^22^ and were kindly provided by Dr. Kefei Yu (Michigan State University). Derivatives of the *LIG1* null cells stably expressing either wild type LigI or the non-methylatable TARK mutant version of LigI were generated as previously described^15^. Above mentioned cell lines were maintained in RPMI Medium 1640 supplemented with 10% FBS, 1% penicillin/streptomycin, and freshly added 55 µM β-mercaptoethanol at 37 °C in humidified atmosphere with 5% (v/v) CO_2_. 46BR.1G1 cells a complemented derivative stably expressing wild type LigI (7A3) were maintained in DMEM F-12, 10% FBS, 1% penicillin/streptomycin, 2 mM glutamine, 0.5 mg/ml G418 at 37 °C in humidified atmosphere with 5% (v/v) CO_2_^25,26,32^. Independent isolates of the 46BR.1G1 strain stably transfected with the empty expression vector and a complemented derivative stably expressing wild type LigI (7A3) were kindly provided by Dr. Alessandro Montecucco^31^. SV40-transformed human fibroblasts MRC-5V1 and GM00847 expressing endogenous wild type LigI were maintained in the same medium without G418^25,26,31,32^.

### Primary antibodies

Anti-PCNA (Santa Cruz, cat. no. sc-56, 1:200), anti-GAPDH (Cell Signaling, cat.no. 2118S, 1:1000), anti-H4 (Abcam, cat.no. ab17036, 1:1000), anti-Lig1 (1:5000), anti-actin (Cell Signaling, cat.no. 12620S, 1:1000), anti-XRCC1 (Santa Cruz, cat.no. sc-56254, 1:200), anti-LIGIIIα (GeneTex, cat.no. GTX103172, 1:1000), anti-DNMT1 (Abcam, cat.no. ab19905, 1:1000), anti-UHRF1 (Abcam, cat.no. ab57083, 1:500), anti-H1.0 (Abcam, cat.no. ab11079, 1:500) and anti-H4 (Abcam, cat.no. ab17036, 1:1000) antibodies were used for immunoblotting.

### Cell lysates and subcellular fractionation

For whole cell lysate generation, cells were gently washed in PBS and resuspended in 250 μl ice-cold RIPA buffer supplemented with 1× protease inhibitor cocktail (Sigma-Aldrich, cat.no. P8340) and 1 mM PMSF. Samples were then sonicated on ice (2 rounds of 10’’ on and 10’’ off cycles at 10 W output) to shear the DNA, followed by centrifugation for 10 min at 16,400×g at 4 °C. Clarified lysate (200 μl) was mixed with 2× Laemmli buffer (with 5% v/v β-mercaptoethanol) prior to heating for 5 min at 95 °C. Subcellular fractionation was performed using the subcellular protein fractionation kit for cultured cells (Thermo Fisher Scientific, cat. no. 78840) according to the manufacturer’s instructions. Protein concentrations were determined by using the assay described by Bradford^59^ (Bio-Rad, cat. no. 500-0006). Proteins (50 µg) in the whole cell lysates or chromatin fraction were separated on 12% SDS polyacrylamide gels and transferred onto Immuno-blot LF PVDF membranes (Bio-Rad, cat. No. 162-0261). After transfer, membranes were blocked using Odyssey NIR blocking buffer (1:3 in TBS) (Li-Cor, cat. No. 927-50000) for 1 h at room temperature. Subsequently, membranes were incubated overnight at 4 °C with anti-H1.0 (Abcam, cat.no. ab11079, 1:500) and anti-H4 (Abcam, cat.no. ab17036, 1:1000). This was followed by 4 × 5 min washes with TBST and a final wash with 1× TBS. After this the membranes were incubated with secondary antibodies, Goat anti-Mouse IRDye 680RD (Li-Cor, cat. no. 925-68070, 1:5000) or Goat anti-mouse IRDye 800CW (Li-Cor, cat.no. 925-32210, 1:5000) for 1 h at room temperature protected in the dark prior to imaging at 680 nm and 800 nm, respectively using an Odyssey NIR imaging system. Images were analyzed using Image Studio Lite Ver 5.2.

### aniPOND (accelerated native isolation of protein on nascent DNA)

aniPOND was performed as described previously^24^. Briefly, CH12F3 cells were grown in T175 flasks for 48 h until approximately 1 × 10^8^ cells were present in exponential growth phase. Cells were pulse labelled with 10 μM EdU (Sigma-Aldrich, cat. no. T511285) for 15 min followed by centrifugation and aspiration of the EdU-containing medium. No-click control (NCC) and pulse (T0) samples were resuspended in ice cold buffer A (20 mM HEPES-NaOH pH 7.2, 50 mM NaCl, 3 mM MgCl_2_, 300 mM sucrose, 0.5% IGEPAL CA630) and thymidine chase samples were resuspended in thymidine (Sigma-Aldrich, cat. no. T9250) containing medium to a final concentration of 10 µM for indicated chase times lengths following which the chase samples were also resuspended in buffer A to lyse the cells and obtain the nuclei. The nuclei were then washed with ice-cold PBS and treated with Click /No click reaction mixture (25 μM biotin-PEG3-azide; Sigma-Aldrich, cat.no. 762024 (DMSO used for NCC), 10 mM sodium l-ascorbate, 2 mM copper (II) sulfate in PBS). Following an ice-cold wash in phosphate-buffered saline, nuclei were treated with buffer B (25 mM NaCl, 2 mM EDTA, 50 mM Tris–HCl pH 8.0, 1% IGEPAL CA630, protease inhibitors) and subjected to sonication on ice (6 rounds of 10’’ on and 10’’ off cycles at 10W output). The sonicated chromatin fraction (20 µl) was removed to be used as INPUT. Streptavidin-coated beads (Pierce, cat. No. 20359) were added to the rest of the chromatin fraction and rotated at 4 °C overnight to immunoprecipitate the biotin labelled chromatin. The sample was extensively washed with Buffer B without protease inhibitors and all of the buffer was aspirated after the last wash. The beads were resuspended in 2× Laemmli buffer with 5% v/v β-mercaptoethanol and boiled for 15 min to elute the bead bound proteins. The input and capture samples were separated by SDS-PAGE gel and proteins were detected by immunoblotting using NIR fluorophore labeled secondary antibody conjugates.

Identification of proteins by mass spectrometry following aniPOND was performed as described previously^60,61^. Briefly, CH12F3-WT and *LIG1* null cells were grown either in light or heavy SILAC media and processed by aniPOND as described earlier except that prior to the click reaction, samples from light and heavy labeled cells were mixed 1:1 and then separated by SDS-PAGE and stained with Coomassie blue*.* Gel regions above and below the streptavidin band were excised and then processed as described^60,61^. Tryptic peptides were separated by MudPIT using an 8-step salt gradient and then identified by mass spectrometry as described (MS) in the Vanderbilt Mass Spectrometry Research Center Proteomics Core^60,61^. For peptide and protein identification, data were analyzed using the Maxquant software package, version 1.3.0.5 with MS/MS spectra searched against the UniprotKB protein database. Peptide intensities for specific proteins were normalized by dividing by the intensity value for H4 histone peptides. The ratio of the normalized values for a protein were calculated by the dividing normalized values from the heavy isotope-labeled extract from the* LIG1* null cells by the normalized values from the light isotope-labeled extract from the wild type parental cells (Run 1). Similar experiments were carried out in which the heavy and light isotope media were switched (Run 2). For the analysis of bulk chromatin, the arithmetic mean of the ratios for specific proteins from the run 1 and run 2 were calculated used as the average ratio shown on the plots. The calculations and graphics were performed by programming in R 4.0.1^62^ using the free platform RStudio 2022.02.03^63^.

## Supplementary Information

Supplementary Information.

## Data Availability

The mass spectrometry proteomics data have been deposited to the ProteomeXchange Consortium via the PRIDE partner repository with the dataset identifier PXD039547 (Reviewer account details: username: reviewer_pxd039547@ebi.ac.uk; Password: GVOqVmgp). All the gene expression data has been deposited at NCBI’s SRA database under the accession number PRJNA918366 (Reviewer’s link: https://dataview.ncbi.nlm.nih.gov/object/PRJNA918366?reviewer=kd6ibsar33vl6t8slv5e93fm9t).
