# Supplementary material for: Unchanged PCNA and DNMT1 dynamics during replication in DNA ligase I-deficient cells but abnormal chromatin levels of non-replicative histone H1
Source: Sci Rep. 2023 Mar 16;13:4363. doi: 10.1038/s41598-023-31367-4 (PMC10020546; doi:10.1038/s41598-023-31367-4)

SUPPLEMENTAL MATERIAL

Title: Unchanged PCNA and DNMT1 dynamics during replication in DNA ligase I-deficient cells but abnormal chromatin levels of non-replicative histone H1

Authors: Seema Khattri Bhandari, Nathaniel Wiest, Annahita Sallmyr, Ruofei Du, Laure Ferry, Pierre-Antoine Defossez, and Alan E. Tomkinson

Methods

RNA Isolation and Sequencing: Total RNA was extracted using RNeasy micro kit (Qiagen, cat.no. 74004). Synthesis of cDNA and library preparation were performed using the SMARTer Universal Low Input RNA Kit for Sequencing (Clontech) and the Ion Plus Fragment Library Kit (ThermoFisher) as previously described [1-3]. Sequencing was performed using the Ion Proton S5/XL systems (Life Technologies) in the Analytical and Translational Genomics Shared Resource at the University of New Mexico Comprehensive Cancer Center.

Standard analysis: Sequences were aligned using TMAP (v4.0.6) to a BED file containing non-overlapping exons from UCSC genome mm10. Gene counts were calculated using HT-Seq with the Feature Counter plugin (v1.0.6, mode = union). Samples were normalized for library size using edgeR and low expressing genes were excluded from the final analysis using a filtering threshold of 10 reads in 2 samples. edgeR and DESeq were used for principal component analysis. edgeR was also used for the differential expression (crosswise comparison of the three groups) using the glm method with an adjusted p-value of cutoff of < 0.05, and requiring a minimum fold change of 2.0. Differentially expressed genes were further analyzed using various R packages including topGO (v2.26.0), gage (v2.24.0) and pathview (v1.14.0) [3-5].

References

[1] K.J. Brayer, C.A. Frerich, H. Kang, S.A. Ness, Recurrent Fusions in MYB and MYBL1 Define a Common, Transcription Factor-Driven Oncogenic Pathway in Salivary Gland Adenoid Cystic Carcinoma, Cancer Discov, 6 (2016) 176-187.
[2] C.A. Frerich, K.J. Brayer, B.M. Painter, H. Kang, Y. Mitani, A.K. El-Naggar, S.A. Ness, Transcriptomes define distinct subgroups of salivary gland adenoid cystic carcinoma with different driver mutations and outcomes, Oncotarget, 9 (2018) 7341-7358.
[3] R.B. Brown, N.J. Madrid, H. Suzuki, S.A. Ness, Optimized approach for Ion Proton RNA sequencing reveals details of RNA splicing and editing features of the transcriptome, PLoS One, 12 (2017) e0176675.
[4] W. Luo, C. Brouwer, Pathview: an R/Bioconductor package for pathway-based data integration and visualization, Bioinformatics, 29 (2013) 1830-1831.
[5] A. Alexa, J. Rahnenfuhrer, T. Lengauer, Improved scoring of functional groups from gene expression data by decorrelating GO graph structure, Bioinformatics, 22 (2006) 1600-1607.

Figure Legends

Fig S1. Absence of LigI and its effect on proteins kinetics on replicating DNA. The relative levels of; **(a)** LigI; **(b)** PCNA; **(c)** ATAD5; **(d)** RFC1; **(e)** RFC2; **(f)** RFC3; **(g)** RFC4; **(h)** RFC5; **(i)** Pol δ**; (j)** Pol ε; **(k)** DNMT1; **(l)** UHRF1; **(m)** G9A; **(n)** GLP; and **(o)** XRCC1 associated with EdU-labeled DNA in

CH12F3 wild type cells (WT:L, blue line) and a *LIG1* null derivative (LigI:H, red line) were determined by mass spectrometry as described in Materials and Methods.

Fig S2. Absence of LigI and its effect on protein association with bulk chromatin. SILAC-MS data showing the levels of proteins in the bulk chromatin as a ratio of *LIG1* null to WT cells in two independent experiments (red square and blue triangle) and their average (grey dot).

Fig S3. Total steady state levels of LigIIIα, XRCC1 and H1.0 in whole cell extracts from CH12F3 wild type and *LIG1* null derivatives. Immunoblots of whole cell extracts (40 µg) from CH12F3 wild type cells (WT) and a *LIG1* null derivative (*LIG1* null) with the indicated antibodies.

Fig S4. Ligase I deficiency does not alter histone protein H4 deposition on replicating DNA. (a) CH12F3 WT, *LIG1-/-* mouse B cells were processed by aniPOND and immunoblotted for H4. CAP = 50% of beads capture proteins. INP = 1% of pre-pulldown proteins. T0, T30, and T60 = 15 min EdU pulse followed by 0, 30, and 60 min thymidine chase respectively. The relative levels of; **(b)** H1.0; **(c)** H2AX; and **(d)** H3.3; associated with EdU-labeled DNA in CH12F3 wild type cells (WT: L, blue line) and a *LIG1* null derivative (LigI:H, red line) were determined by mass spectrometry as described in Materials and Methods. Uncropped Western blots are shown in supplementary Fig S10.

Fig S5. Levels of chromatin bound H1.0 in SV40 immortalized human fibroblasts. Immunoblots of chromatin fractions (50 µg) from human LigI deficient fibroblast cell line 46BR.1G1 (VC), a derivative stably expressing wild type LigI (WTC), and a Simian Virus 40 transformed human skin fibroblast cell line, GM00847 with the indicated antibodies.

Table S1. Gene expression analysis: Expression of various genes was compared between two sets of Lig1 null and WT cells. Fold change value for each gene expression level is calculated as a ratio between Lig1 null to WT cells and a value of <1 or >1 indicates higher gene expression in WT or Lig1 null cells respectively.

Fig S6. Uncropped Western blot images for Figure 1: The samples were run on two different gels (12%) to blot for INPUT and CAPTURE using samples derived from same experiment, blots were processed and imaged in parallel. White boxes indicate the region cropped for final representation. **(a)** blots used in Figure 1b; **(b)** blots used in Figure 1d.

Fig S7. Uncropped Western blots for Figure 4: Subcellular fractionation samples derived from same experiment were run on 7.5% (LigIIIα, XRCC1) and 12% (H4) gels. Blots were cropped to stain for multiple proteins and were processed and imaged in parallel under similar conditions, repeat #3 used for representative purpose in fig 4c, repeat #2 was used for Fig 4d. White boxes indicate the region used for final representation.

Fig S8. Uncropped Western blots for Figure 5: Subcellular fractionation samples derived from same experiments were run on 7.5% (DNMT1, UHRF1) and 12% (H4) gels which were processed and imaged in parallel under similar conditions. This experiment is similar to repeat#2 blot in Figure 4 as the same extracts were used to stain for multiple proteins by cutting the blots. White boxes indicate the region used for final representation.

Fig S9. Uncropped Western blots for Figure 7: (a) Each biological replicate was run on the same gel and processed similarly, repeat #2 used for representative purpose in Figure 7a; **(b)** high-contrast images for Fig S9 (a) showing ladder and blot edges; **(c)** Each biological replicate was run on the same gel and processed similarly repeat #1 used for representative purpose in Figure 7b.

Fig S10. Uncropped Western blots for Figure S4: (a) Samples derived from same experiment were run on two separate gels (12%) to blot for INPUT and CAPTURE, blots were processed and imaged in parallel. White boxes indicate the region cropped for final representation; **(b)** high-contrast image to show ladder and blot edges.

Fig S11. Uncropped Western blots for Figure S5: (a) Samples derived from same experiment were run on gels (12%) to blot for indicated antibodies.

Supplemental Figures:

Figure S1.

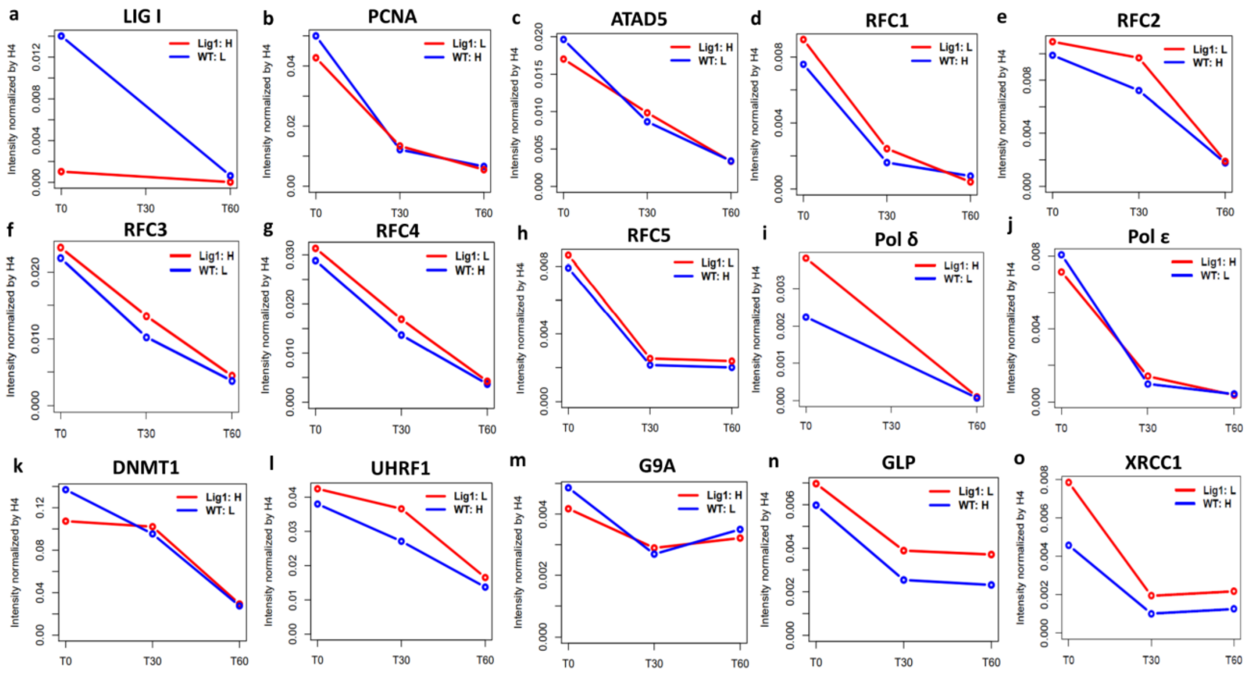

Figure S2.

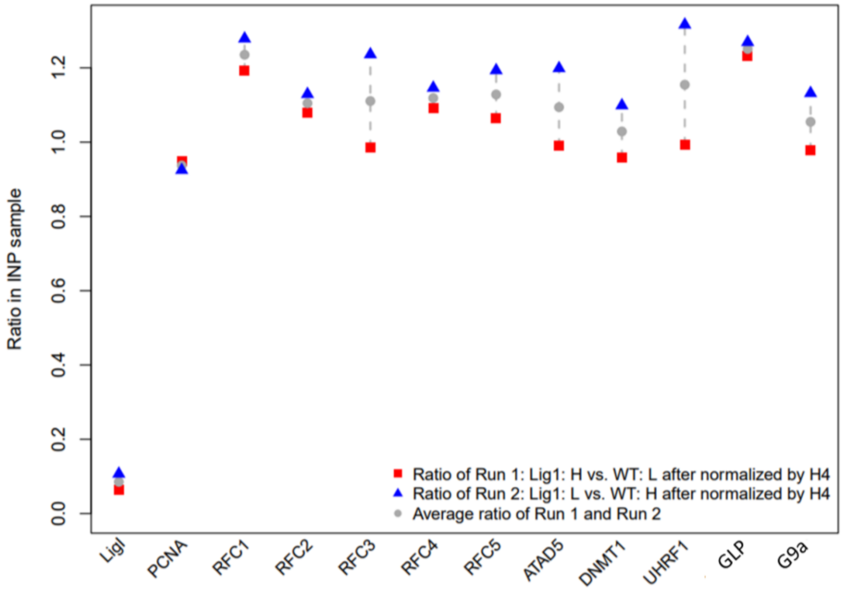

Figure S3.

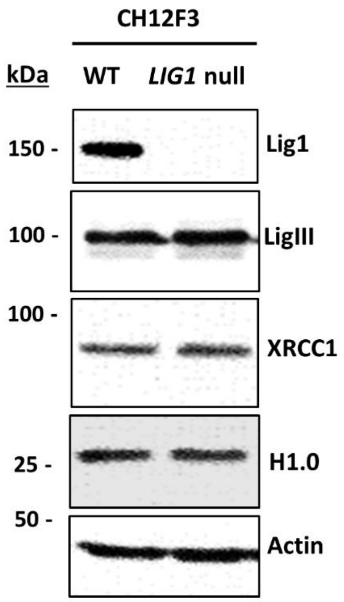

Figure S4.

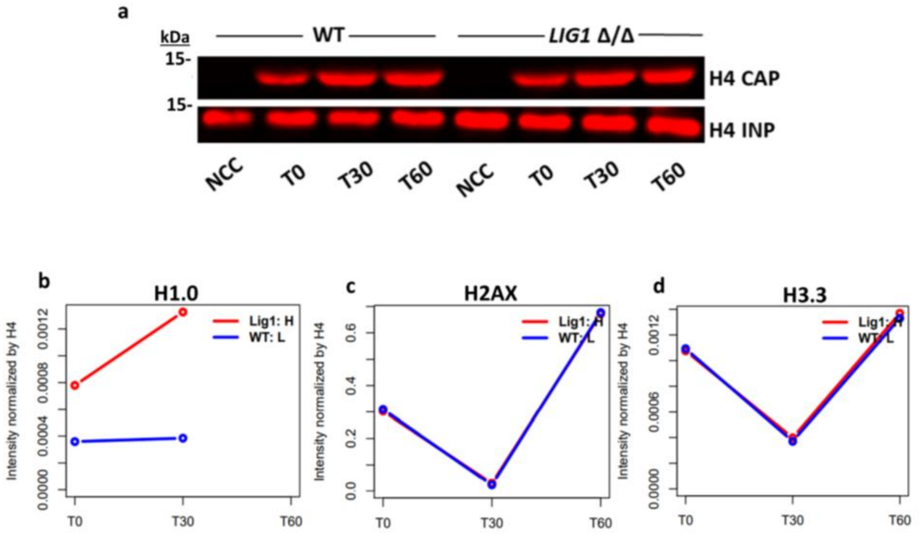

Figure S5.

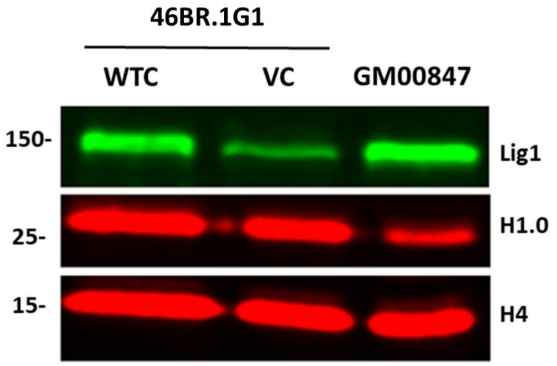

Table S1.

Gene	LIG1 null#1	LIG1 null#2	Wild type #1	Wild type #2	log2 fold change	Fold change	AdjPVal
Lig1	195	185	1036	1112	2.52	0.17	3.84E-46
Pcna	606	740	868	737	0.28	0.82	0.460339
Atad5	659	628	610	599	-0.06	1.05	0.921922
Rfc1	889	871	735	976	-0.02	1.01	0.986527
Rfc2	435	435	400	431	-0.04	1.03	0.961347
Rfc3	89	108	113	114	0.23	0.85	0.735113
Rfc5	682	685	616	474	-0.3	1.23	0.401741
Pold1	551	491	459	428	-0.21	1.15	0.623993
Pold2	974	1072	995	865	-0.11	1.08	0.838291
Pold3	756	680	682	733	0	1	0.997085
Pole	1230	1162	968	1085	-0.2	1.14	0.616755
Lig3	653	731	699	587	-0.08071143	1.057539412	0.906313
Xrcc1	1087	800	820	1178	0.108864682	0.92731752	0.886832
Dnmt1	4451	4591	4670	4470	0.04	0.97	0.960074
Uhrf1	3112	3077	2648	2141	-0.34	1.27	0.241831
Ehmt2	735	776	837	777	0.12	0.92	0.813157
H1f0	287	250	109	217	-0.7	1.62	0.082855
Hist2h2ab	110	85	83	90	-0.15	1.11	0.876512
H2afx	602	397	732	441	0.26	0.83	0.702189
H2afz	2757	3016	2929	2178	-0.15	1.11	0.789532
Hist1h1a	1391	1284	1355	1694	0.21	0.86	0.599012
Hist1h1b	1068	784	902	1172	0.19	0.88	0.717968
Hist1h1c	734	460	560	992	0.41	0.76	0.471442
Hist1h1d	1590	1175	1130	1721	0.07	0.95	0.939241
Hist1h1e	1206	989	996	1232	0.05	0.97	0.954066
Hist1h2af	107	52	74	98	0.14	0.91	0.914823
Hist1h2ao	177	109	138	189	0.22	0.86	0.775699
Hist1h2bb	163	129	176	141	0.15	0.9	0.846719
Hist1h2bk	202	211	185	208	-0.05	1.03	0.962275
Hist1h2bm	464	422	394	612	0.21	0.87	0.722576
Hist2h2ac	669	527	594	763	0.21	0.87	0.666225
Hist2h2bb	2508	2216	2854	2196	0.12	0.92	0.832282
Hist2h3b	156	147	172	155	0.14	0.91	0.844102

Figure S6.

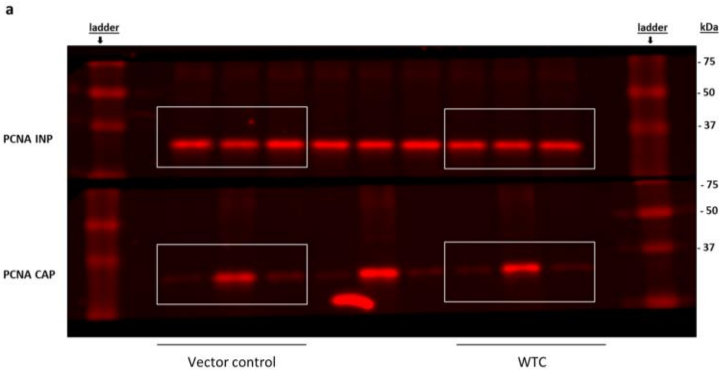

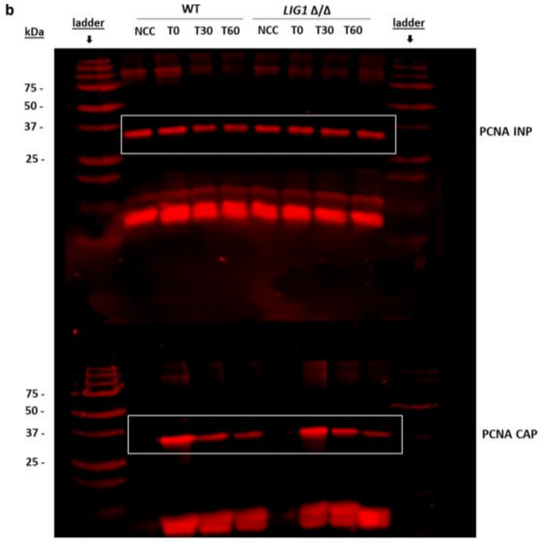

Figure S7.

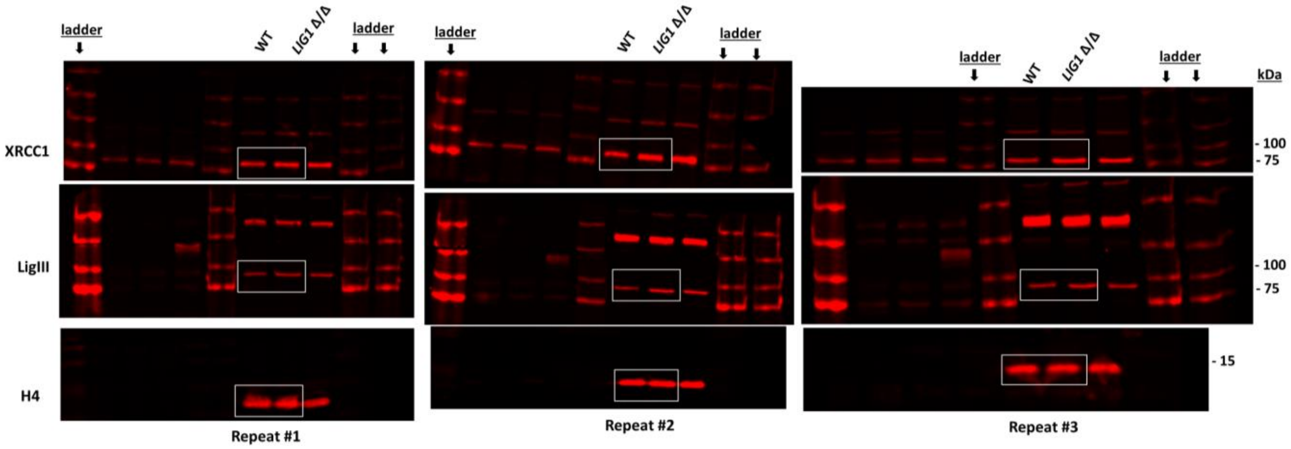

Figure S8.

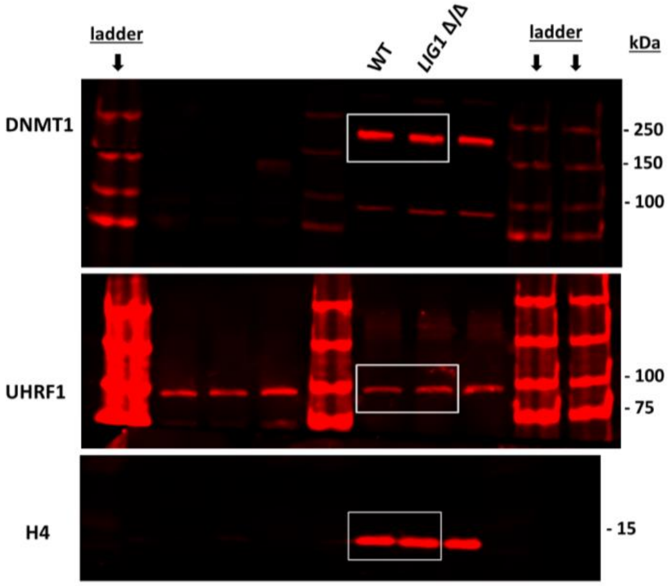

Figure S9.

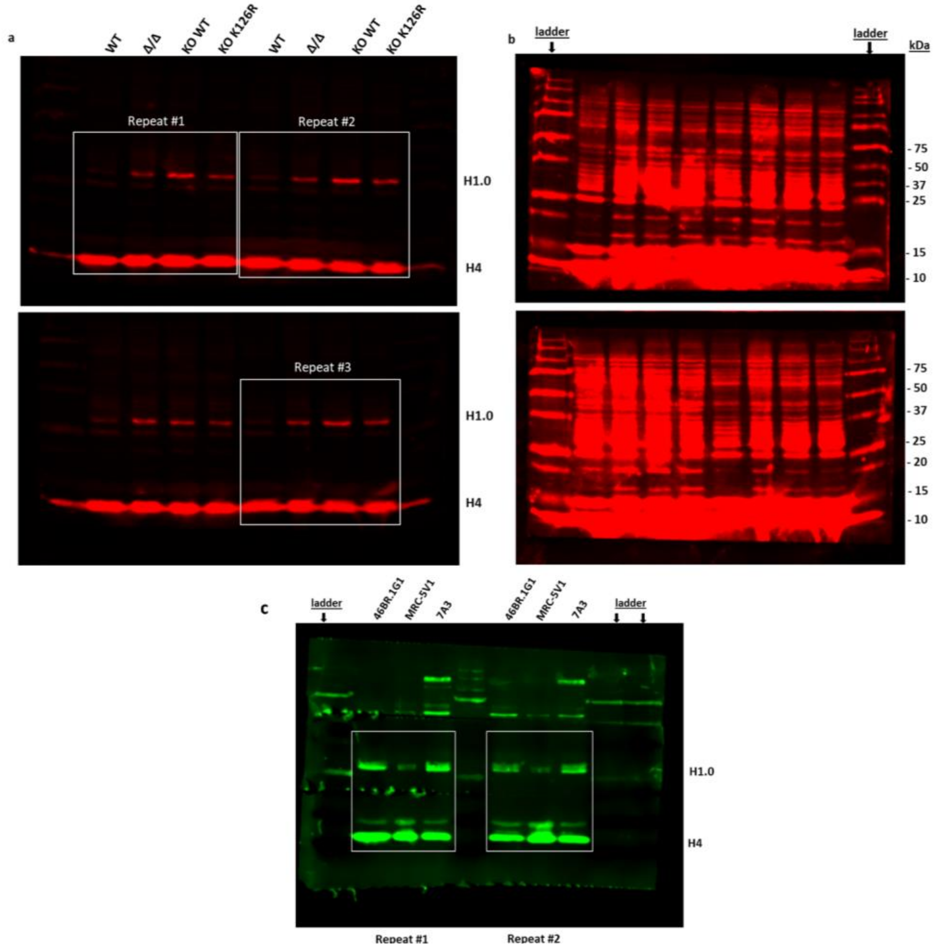

Figure S10.

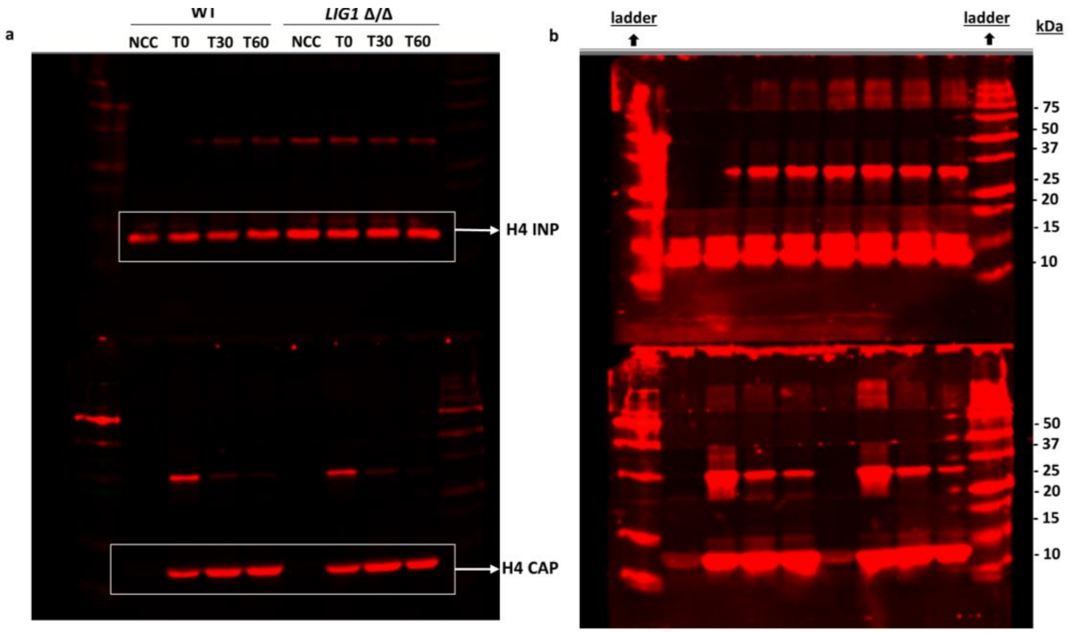

Figure S11.

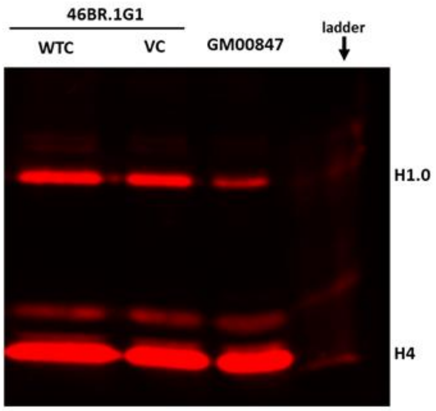

Supplement: Supplementary file 1 — Supplementary Information. [file 41598_2023_31367_MOESM1_ESM.pdf]
